# Combined use of anti-ErbB monoclonal antibodies and erlotinib enhances antibody-dependent cellular cytotoxicity of wild-type erlotinib-sensitive NSCLC cell lines

**DOI:** 10.1186/1476-4598-11-91

**Published:** 2012-12-12

**Authors:** Andrea Cavazzoni, Roberta R Alfieri, Daniele Cretella, Francesca Saccani, Luca Ampollini, Maricla Galetti, Federico Quaini, Gallia Graiani, Denise Madeddu, Paola Mozzoni, Elena Galvani, Silvia La Monica, Mara Bonelli, Claudia Fumarola, Antonio Mutti, Paolo Carbognani, Marcello Tiseo, Elisabetta Barocelli, Pier Giorgio Petronini, Andrea Ardizzoni

**Affiliations:** 1 Department of Clinical and Experimental Medicine, University of Parma, Parma, Italy; 2 Department of Surgical Science, University of Parma, Parma, Italy; 3 Italian Workers’ Compensation Authority (INAIL) Research Center, University of Parma, Parma, Italy; 4 Division of Medical Oncology, University Hospital of Parma, Parma, Italy; 5 Department of Pharmacy, University of Parma, Parma, Italy

**Keywords:** Lung cancer, EGFR, Erlotinib, Cetuximab, ADCC

## Abstract

**Background:**

The epidermal growth factor receptor (EGFR) is an established target for anti-cancer treatment in different tumour types. Two different strategies have been explored to inhibit this pivotal molecule in epithelial cancer development: small molecules TKIs and monoclonal antibodies. ErbB/HER-targeting by monoclonal antibodies such as cetuximab and trastuzumab or tyrosine-kinase inhibitors as gefitinib or erlotinib has been proven effective in the treatment of advanced NSCLC.

**Results:**

In this study we explored the potential of combining either erlotinib with cetuximab or trastuzumab to improve the efficacy of EGFR targeted therapy in EGFR wild-type NSCLC cell lines. Erlotinib treatment was observed to increase EGFR and/or HER2 expression at the plasma membrane level only in NSCLC cell lines sensitive to the drug inducing protein stabilization. The combined treatment had marginal effect on cell proliferation but markedly increased antibody-dependent, NK mediated, cytotoxicity *in vitro*. Moreover, in the Calu-3 xenograft model, the combination significantly inhibited tumour growth when compared with erlotinib and cetuximab alone.

**Conclusion:**

Our results indicate that erlotinib increases surface expression of EGFR and/or HER2 only in EGFR-TKI sensitive NSCLC cell lines and, in turns, leads to increased susceptibility to ADCC both *in vitro* and in a xenograft models. The combination of erlotinib with monoclonal antibodies represents a potential strategy to improve the treatment of wild-type EGFR NSCLC patients sensitive to erlotinib.

## Background

The epidermal growth factor receptor (EGFR, ErbB1, HER1) is the prototypic member of the ErbB family of receptor tyrosine kinases (TKs), which further consists of ErbB2-4 (HER2-4). The ErbB receptors share a similar protein structure, consisting of an extracellular ligand binding domain, a single transmembrane domain and an intracellular C-terminal domain with tyrosine kinase activity [1]. Upon specific binding of EGF-like ligands to the extracellular domain, ErbB receptors dimerize, either as homo- or heterodimers, and undergo autophosphorylation at specific tyrosine residues within the intracellular domain. The phosphorylated tyrosines serve as docking sites for adapter molecules, such as Grb2 and the p85 subunit of PI3K, which activate a complex downstream network. The activated signaling pathways, including the Ras/MAPK, Akt/mTOR kinase and STAT cascades, in turn regulate transcription factors and other proteins involved in cell proliferation, survival, motility and differentiation [2]. Two main strategies targeting ErbB receptors have been developed: small-molecule inhibitors of the tyrosine kinase domain (EGFR tyrosine kinase inhibitors [TKIs], such as erlotinib and gefitinib), and monoclonal antibodies (such as cetuximab, anti-EGFR and trastuzumab, anti-HER2), directed against the extracellular domain, which inhibit phosphorylation/activation and promote internalization. EGFR and HER2 are overexpressed in 40-80% and 25-30%, respectively, of non-small cell lung cancer (NSCLC) patients and their overexpression has been frequently correlated with a poor prognosis [3,4].

Erlotinib is an effective treatment for NSCLC patients and has been registered as a second and third-line treatment of NSCLC regardless of EGFR mutation status [5].

Gefitinib has been registered for the therapy of advanced NSCLC harbouring activating EGFR mutations in the tyrosine kinase domain, the most frequent being L858R in exon 21 and Del (746–750) in exon 19 [6]. Although mutations in EGFR are useful predictors for the activity of EGFR-TKI, they cannot be used as the only criterion to determine who should receive anti-EGFR therapy and it is becoming increasingly clear that even patients with EGFR wild-type can benefit from EGFR-TKI [5,7,8].

Cetuximab is a chimeric IgG1 monoclonal antibody (mAb) that blocks ligand binding to EGFR, leading to a decrease in receptor dimerization, autophosphorylation, and activation of signaling pathways [9]. In addition the binding of cetuximab initiates EGFR internalization and degradation which leads to signal termination. Moreover, unlike EGFR-TKIs, cetuximab can induce antibody dependent cellular cytotoxicity (ADCC) activity, an important immunologic antitumour effect. Cetuximab in combination with chemotherapy has been approved by the FDA for the treatment of metastatic colorectal cancer and of locally advanced head and neck cancer.

Two randomized phase III trials in NSCLC patients, evaluating cetuximab in addition to first-line chemotherapy, showed a small benefit in overall survival for the experimental treatment, which was considered insufficient by the EMA for marketing approval [10,11]. However, a subgroup analysis of the FLEX phase III trial recently demonstrated a larger survival benefit from the experimental treatment in patients with high immunohistochemical EGFR expression [12].

Trastuzumab, registered for the treatment of HER2 positive breast cancer, has also been tested in phase II trials as a single agent and in combination with cytotoxic chemotherapy for patients with NSCLC. These trials have not yet produced any convincing evidence of an improved antitumour activity by adding trastuzumab to standard chemotherapy in NSCLC [13,14].

Several preclinical studies on cell lines from different tumour types, indicated that the association between EGFR/HER2 mAbs with TKIs displays an increased efficacy [15].

In this study we explored the potential of combining erlotinib with either cetuximab or trastuzumab in order to improve the efficacy of EGFR targeted therapy in EGFR wild-type sensitive NSCLC cell lines. Our results indicate that EGFR-TKI increases surface expression of EGFR and/or HER2 only in erlotinib sensitive NSCLC cell lines and, in turns, leads to increased susceptibility to ADCC both *in vitro* and in xenograft models.

## Results

### Differential effects of erlotinib on EGFR and HER2 expression in sensitive and resistant NSCLC cell lines

**Figure 1:**
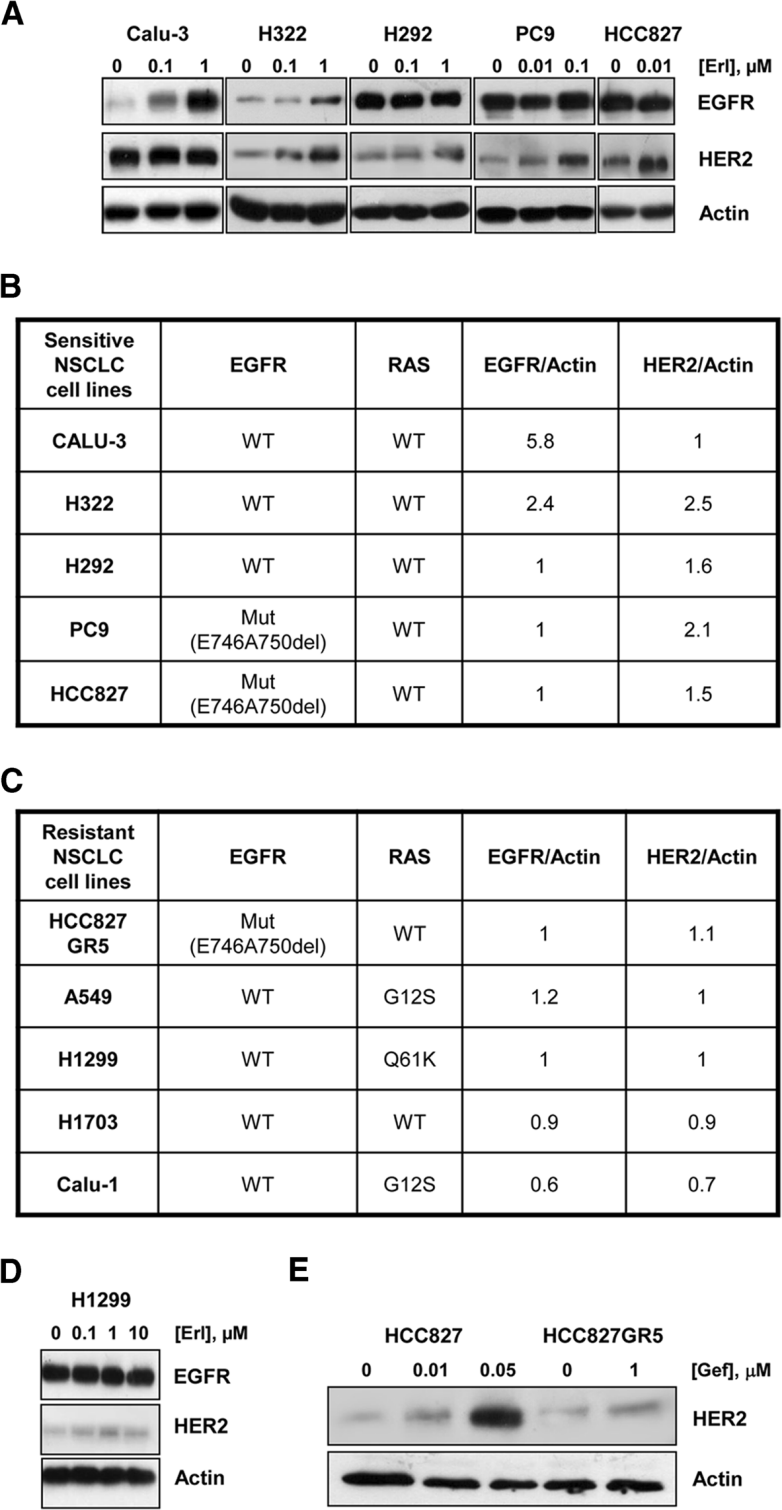
**Erlotinib induces EGFR and HER2 protein accumulation only in sensitive NSCLC cell lines. (A)** Calu-3, H322, H292, PC9 and HCC827 cell lines were treated with the indicated concentrations of erlotinib for 48 h. At the end of the drug treatment cell lysates were immunoblotted to detect the indicated proteins. The immunoreactive spots were quantified by densitometric analysis, ratios of EGFR/Actin and HER2/Actin were calculated at 1 μM erlotinib for Calu-3 H322 and H292 or 10 nM for PC9 and HCC827 and values are expressed as fold increase versus control **(B)**. **(C)** HCC827GR5, A549, H1299, H1703, Calu-1 cell lines were treated with 1 μM erlotinib for 48 h and at the end of treatment cell lysates were immunoblotted to detect the indicated proteins. The immunoreactive spots were quantified by densitometric analysis, ratios of EGFR/Actin and HER2/Actin were calculated and values are expressed as fold increase versus control. **(D)** Representative Western blotting of resistant H1299 cell line exposed to increased concentration of erlotinib. **(E)** HCC827 parental cell line and HCC827GR5 resistant clone were treated with the indicated doses of gefitinib and processed as above. The results are from representative experiments. Each experiment, repeated three times, yielded similar results.

Firstly, we evaluated the effect of erlotinib on total EGFR and HER2 protein levels in sensitive NSCLC cell lines (Calu-3, H322 and H292 cell lines carrying wild-type EGFR; PC9 and HCC827 carrying EGFR E746-A750del mutation) and in resistant cell lines (A549, H1299, H1703 and Calu-1 intrinsically resistant carrying wild-type EGFR; HCC827GR5 with MET amplification as mechanism of acquired resistance to TKI) [16]. As shown in Figure 1A, erlotinib induced accumulation of EGFR protein in Calu-3 and H322 cells while HER2 accumulated in H322, H292, PC9 and HCC827 cells in a dose-dependent manner. The EGFR/Actin and HER2/Actin ratios obtained after treatment at 1 μM or 10 nM erlotinib were calculated and values expressed as fold differences versus control (Figure 1B). In contrast, EGFR and HER2 protein accumulation was not observed in any cancer cell line with intrinsic resistance to EGFR inhibitors until the concentration of 10 μM. Indeed the ratios EGFR/Actin or HER2/Actin were similar or even lower than those calculated in untreated cells (Figure 1C) and similar results were obtained with gefitinib (not shown). A representative Western blotting of resistant H1299 cell line is reported in Figure 1D.

The different effect of TKIs on HER2 expression between sensitive and resistant NSCLC cell lines was confirmed in the HCC827 parental and in the HCC827GR5 resistant clone treated for 48 h with gefitinib (Figure 1E).

### Erlotinib increases the cell surface expression of EGFR and HER2 in erlotinib sensitive NSCLC cell lines

**Figure 2:**
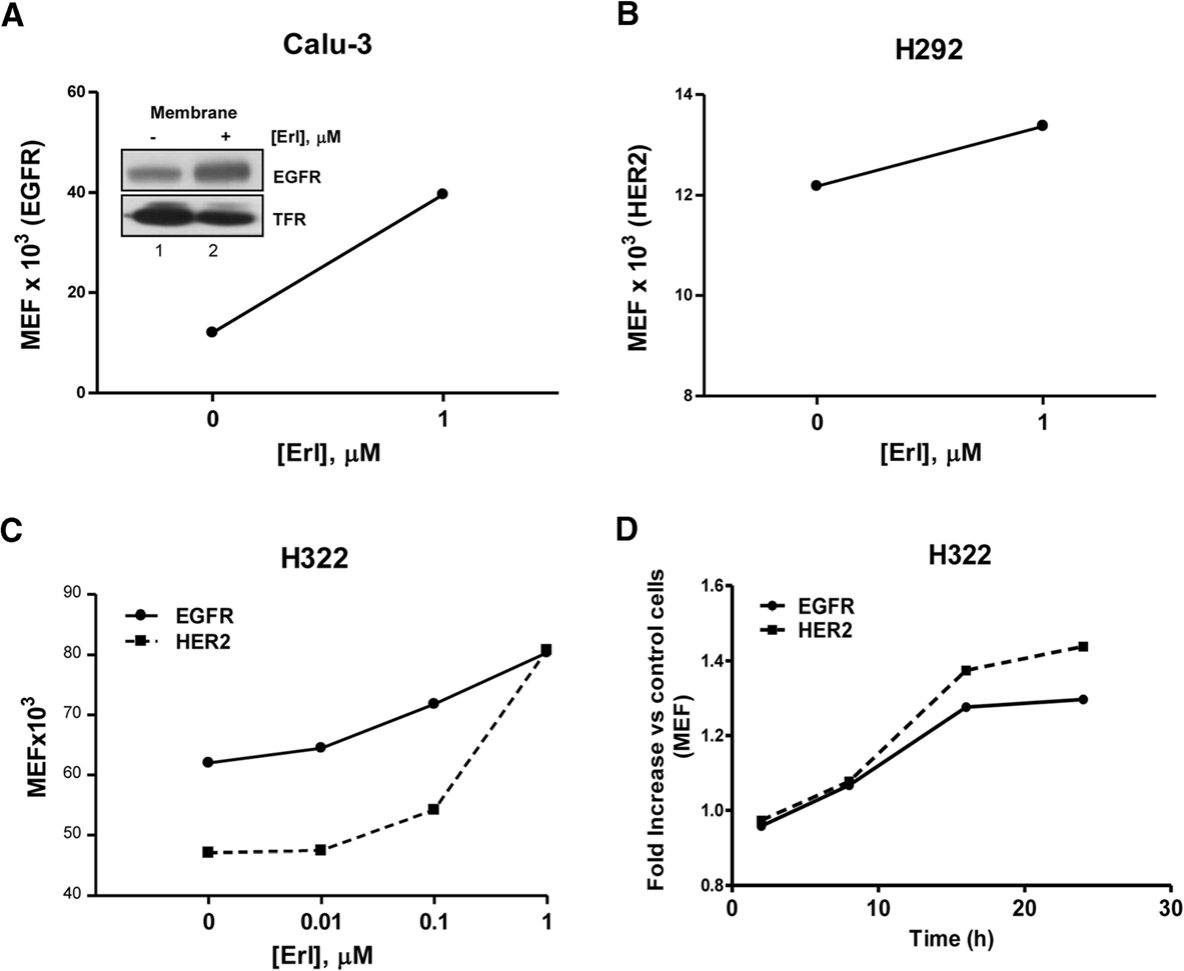
**EGFR and HER2 increase at the plasma-membrane level.** Calu-3 **(A)** and H292 **(B)** cell lines were treated with 1 μM erlotinib for 24 h, H322 cell line was treated with increasing concentration of erlotinib **(C)** or with 1 μM erlotinib for the indicated period of time **(D)**. At the end of the treatment, cell surface expression of EGFR and/or HER2 were evaluated by flow cytometry and the quantification is reported as Molecules of Equivalent Fluorophore [MEF] or as fold increase versus untreated control cells **(D)**. Inset Figure 2A: Western blot analysis of EGFR protein membrane level in Calu-3 after treatment with 1 μM erlotinib for 24 h. Whole cells were labeled with biotin and membrane bound proteins were pulled down with neutrAvidin beads. The results are from representative experiments. Each experiment, repeated three times, yielded similar results.

EGFR and HER2 expression on the plasma membrane was quantified by flow cytometry in sensitive EGFR wild-type NSCLC cell lines Calu-3, H322 and H292 after exposure to 1 μM erlotinib for 24 h. The drug enhanced surface expression, calculated as molecules of equivalent soluble fluorophore, of EGFR in Calu-3 (Figure 2A) and H322 (Figure 2C, 2D) and of HER2 in H292 (Figure 2B) and H322 (Figure 2C, 2D) cell lines. In H322 cell line, the increase in EGFR and HER2 surface expression was dose and time dependent (Figure 2C, 2D). Western blot analysis of isolated cell surface membrane proteins (inset Figure 2A) confirmed the increase of EGFR in erlotinib treated Calu-3 cells.

**Figure 3:**
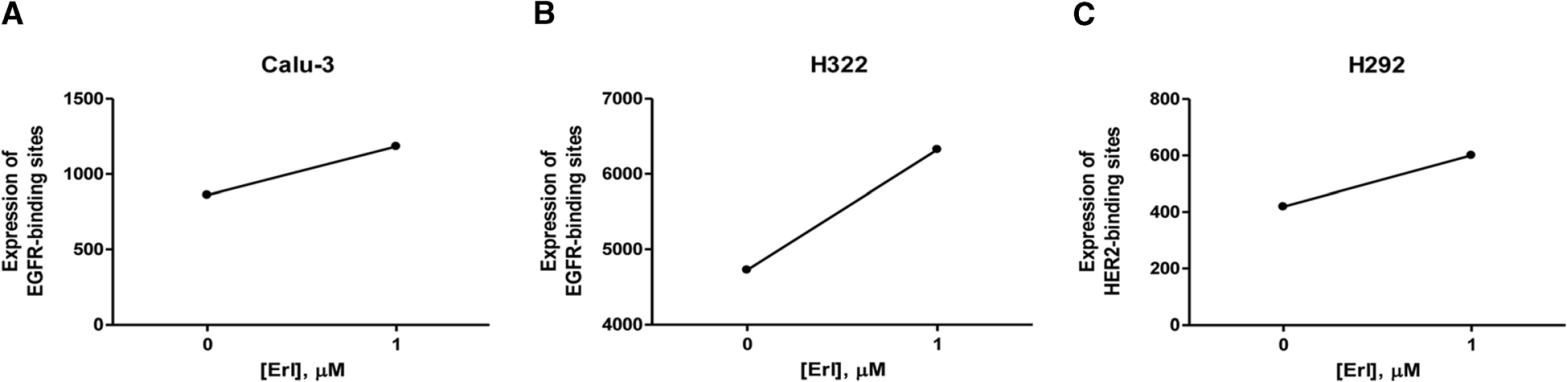
**Erlotinib induces the increase of cetuximab and trastuzumab binding sites.** Calu-3, H322 and H292 cell lines were treated with erlotinib for 24 h. Binding sites were assessed by flow cytometry using cetuximab (Calu-3, H322) and trastuzumab (H292) as primary antibodies followed by PE-anti-human IgG exposure. Binding sites quantification is reported as Molecules of Equivalent Fluorophore [MEF]. The results in **A**, **B**, **C** are from representative experiments. Each experiment, repeated three times, yielded similar results.

Exploiting the ability of cetuximab and trastuzumab to bind EGFR and HER2, we used these mAbs as primary antibodies for flow cytometry analysis. By this approach, as shown in Figure 3, we confirmed that the surface density of cetuximab and trastuzumab-binding sites, respectively, on Calu-3 (Figure 3A), H322 (3B) and H292 (3C) cells were increased after 1 μM erlotinib treatment. These results suggest that erlotinib enhanced cell surface expression of EGFR or HER2 on sensitive NSCLC cells, leading to an increase of mAbs binding to cancer cell surface.

### Erlotinib induces EGFR protein stabilization

**Figure 4:**
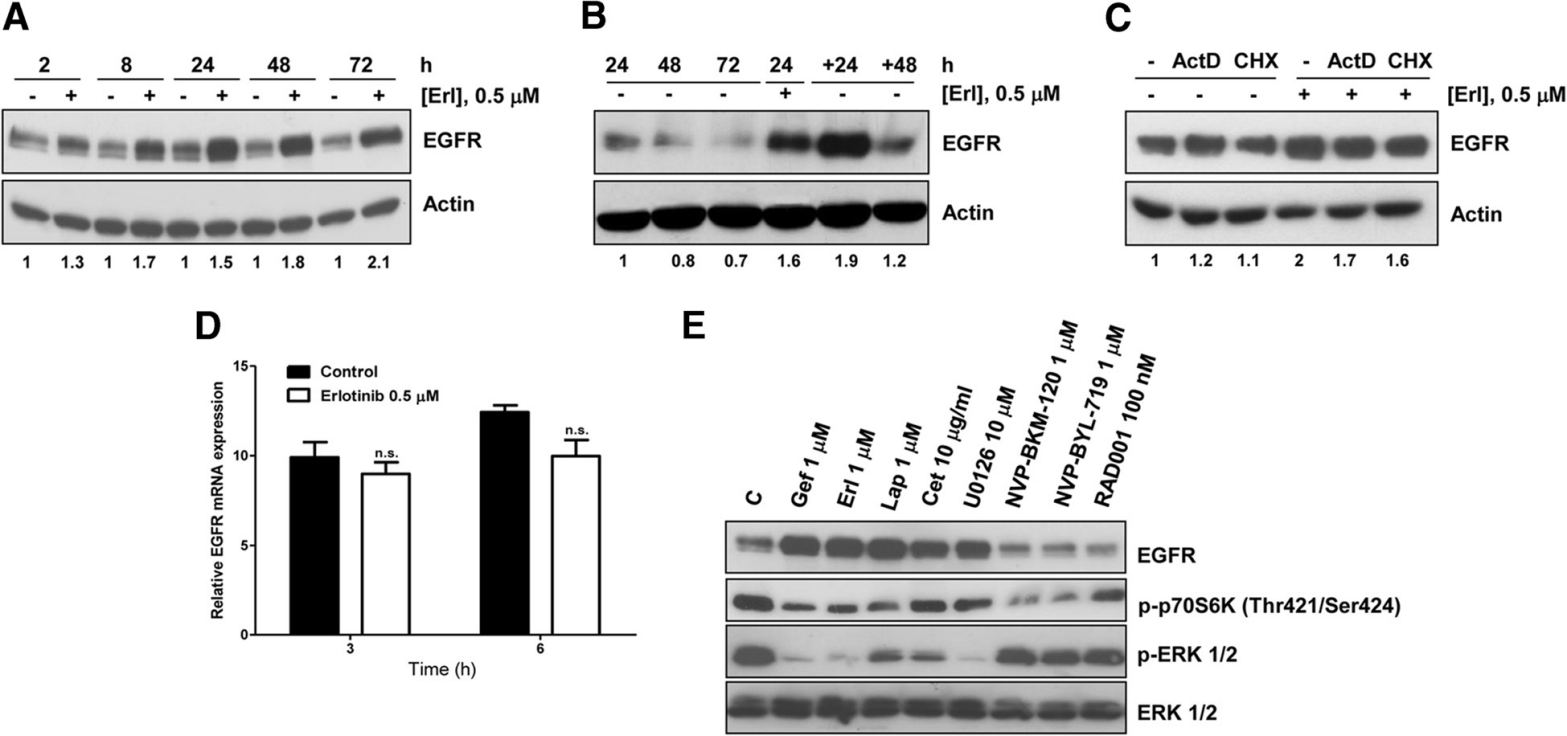
**Erlotinib induces EGFR protein accumulation through protein stabilization. (A)** Calu-3 cells were treated for the indicated period of time with 0.5 μM erlotinib. At the end of drug treatments cell lysates were immunoblotted to detect EGFR protein. **(B)** Calu-3 cells were treated with 0.5 μM erlotinib for 24 h then the drug was removed and drug-free medium was added for further 24 and 48 h. Then, cell lysates were immunoblotted to detect the EGFR protein levels. **(C)** Calu-3 cells were treated for 24 h with erlotinib 0.5 μM, in the absence/presence of 0.1 μg/ml actynomicin D and 2 μg/ml cyclohexymide. At the end of the experiment, cell lysates were immunoblotted to detect the indicated proteins. The immunoreactive spots were quantified by densitometric analysis, ratios of EGFR/Actin were calculated and values are expressed as fold increase versus control. **(D)** Calu-3 cells were exposed to 0.5 μM erlotinib for the indicated period of time and the EGFR mRNA was detected by RT-PCR. The mean values of two independent measurements (± SD) are shown. **(E)** EGFR, p-P70S6K, p-P44/42 and P44/42 were detected by Western blotting in Calu-3 cells untreated or treated for 24 h with 1 μM gefitinib, erlotinib and lapatinib, 10 μg/ml cetuximab, 10 μM U0126, 1 μM NVP-BKM-120 and NVP-BYL-719 and 100 nM RAD001. The results are from representative experiments. Each experiment, repeated three times, yielded similar results.

The possibility that the higher EGFR level observed in Calu-3 cells exposed to erlotinib was due to protein stabilization or increased synthesis was then explored. As shown in Figure 4A, EGFR level increased after 2 h of erlotinib treatment and reached a plateau after 24 h. Furthermore, the maximum level was maintained during time in the presence of the drug. However, after 48 h of erlotinib removal, EGFR expression was reduced to level comparable to untreated cells (Figure 4B). Calu-3 were also treated with erlotinib in the presence of specific inhibitors of mRNA (Actynomicin D) and protein (Cycloheximide) synthesis. As shown in Figure 4C, the erlotinib- induced EGFR protein increase was neither influenced by Actynomicin D nor Cycloheximide treatment indicating that the higher level of EGFR after erlotinib treatment could be ascribed to post-transcriptional mechanisms such as protein stabilization. Moreover, we analyzed EGFR transcript level by real time PCR after erlotinib treatment (Figure 4D). Erlotinib did not affect EGFR mRNA level when compared to untreated cells.

With the aim to clarify why the increased level of EGFR was induced only in sensitive cells, we then tested the effect of EGFR inhibitors (gefitinib, erlotinib, cetuximab, lapatinib) and of inhibitors of MAPK and PI3K/AKT/mTOR signaling transduction pathways on EGFR accumulation in Calu-3 cell line. Gefitinib, erlotinib, lapatinib significantly inhibited the phosphorylation of p70S6K and p44/42 and induced a significant increase in EGFR protein level (Figure 4E). The MEK inhibitor U0126 strongly enhanced EGFR expression, in contrast no increase in the EGFR level was observed after incubation with the inhibitors of PI3K/AKT/mTOR pathway tested (NVP-BKM-120 and NVP-BYL-719 PI3K inhibitors and RAD001 mTOR inhibitor).

### Effects of erlotinib and cetuximab combined treatment on NSCLC cell growth and antibody-dependent cell-mediated cytotoxicity

**Figure 5:**
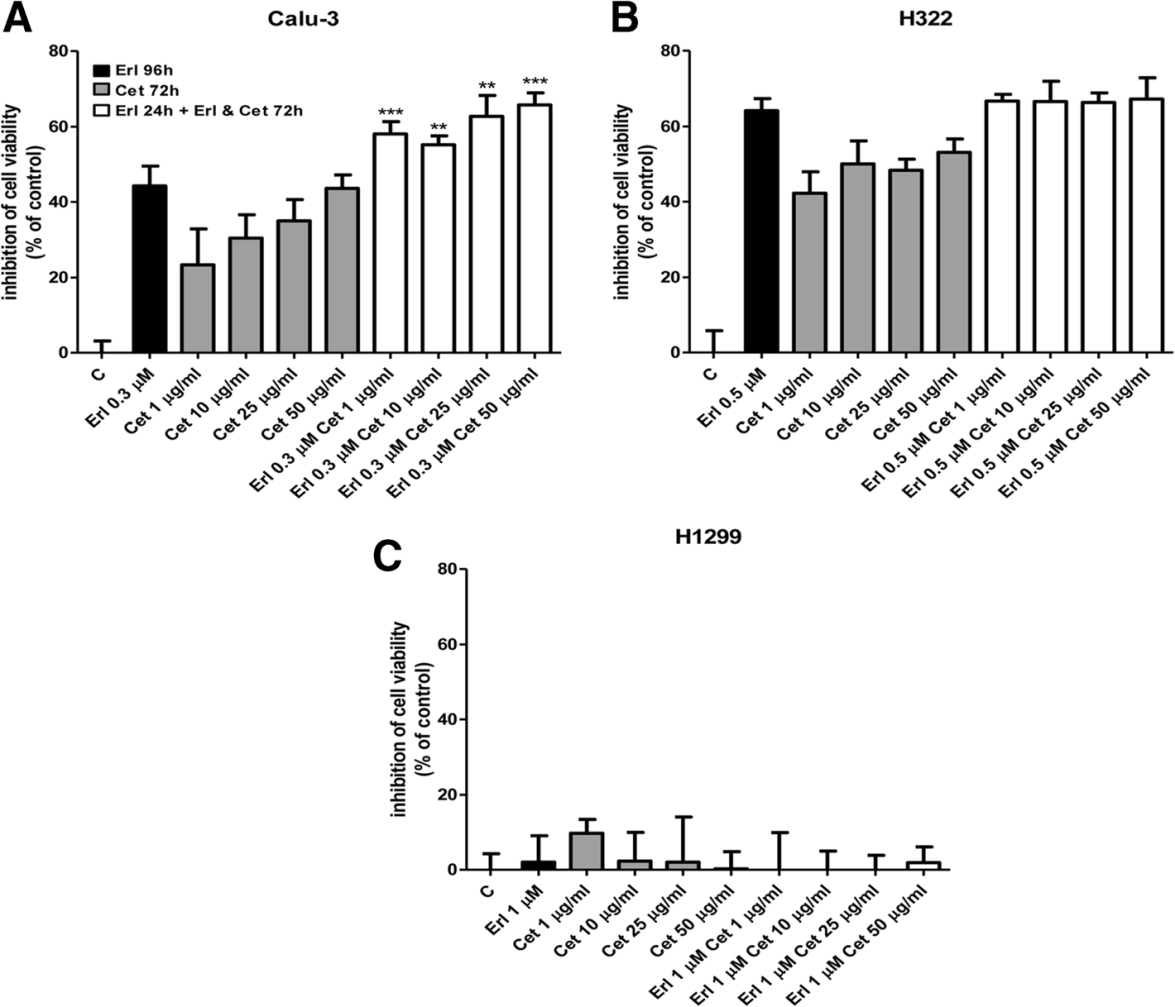
**Effect of erlotinib and cetuximab combination on cell viability in NSCLC cell lines.** Calu-3, H322 sensitive cells **(A**, **B)** and H1299 resistant cells **(C)** were exposed to the indicated concentrations of erlotinib for 96 h or cetuximab for 72 h and to erlotinib for 24 h followed by the combination of erlotinib and cetuximab for 72 h. After the treatments, cell viability was assessed by MTT assay. Data are expressed as percent inhibition of cell viability versus control cells and are mean values of three separate experiments (**P < 0.01 ***P < 0.001).

We then investigated the effect of targeting EGFR by both the TKI erlotinib and the mAb cetuximab in a cell viability assay (Figure 5). We treated Calu-3, H322 and H1299 cells with erlotinib, cetuximab (doses ranged from 1 to 50 μg/ml) or the combination based on the schedule erlotinib 24 h followed by the combination of erlotinib with cetuximab for 72 h. As expected Calu-3 (Figure 5A) and H322 (Figure 5B) cells were responsive to erlotinib and cetuximab treatment, whereas H1299 (Figure 5C) cells were resistant to both the single regimens. Comparing the experimental combination points with that expected by the Bliss criterion, an additive effect was observed only in the Calu-3 cells. In fact, in the H322 cells we failed to observe any improvement treating cells with the combined treatment and H1299 remained resistant.

Moreover, cell death, evaluated by morphological analysis, caspase-3 activation and cleavage, was negligible under any of the tested treatments at all the time points analyzed (not shown) suggesting that the combined erlotinib-cetuximab treatment exerted a cytostatic and not a cytotoxic effect.

**Figure 6:**
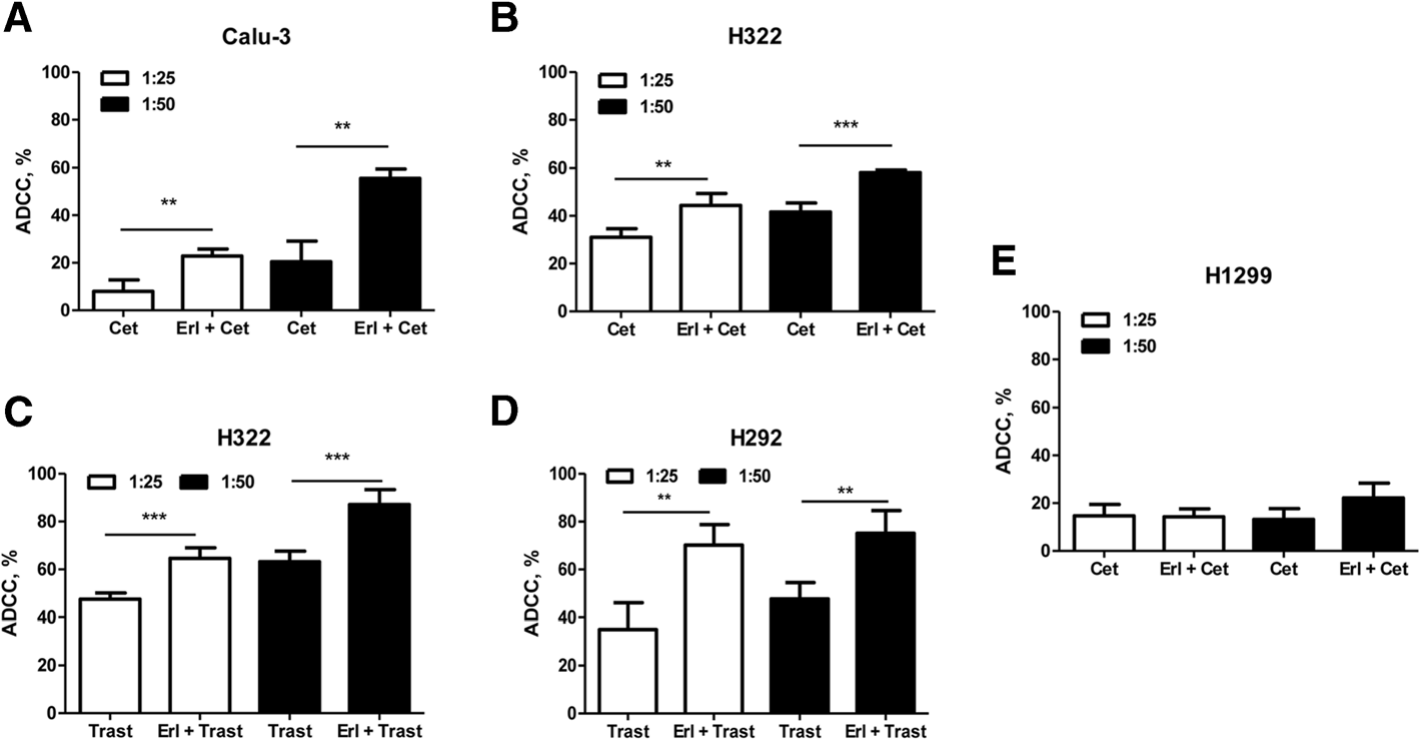
**Erlotinib potentiates antibody dependent cell cytotoxicity.** The indicated human NSCLC cell lines were treated with 1 μM erlotinib for 24 h. After the treatment with erlotinib, 10 μg/ml Cetuximab **(A**, **B**, **E)** or Trastuzumab **(C**, **D)** were added to cancer cells seeded with 100 U/ml IL-2 activated-NK cells at the ratio of 1:25 and 1:50. After 4 h LDH release was determined as described in Methods section. The results are from representative experiments. The experiment, repeated three times, yielded similar results (**P < 0.01 ***P < 0.001).

Since the engagement of immune component system is one of the main mechanisms of the activity of specific mAbs directed to ErbB family members *in vivo*, we examined whether erlotinib could enhance cetuximab- or trastuzumab-mediated ADCC by NK cells. As shown respectively in Figure 6 A-B cetuximab-dependent cytotoxicity in the presence of IL-2 activated NK cells was higher in Calu-3 and H322 cells previously treated with erlotinib compared with cells treated with cetuximab alone. Similarly, trastuzumab-dependent cytotoxicity was higher in H322 and H292 cells (Figure 6 C-D) previously treated with erlotinib compared with cells treated with trastuzumab alone.

On the contrary, the combination of erlotinib with cetuximab did not significantly modify the mAb dependent cytotoxicity in H1299 resistant cancer cells.

### Effect of erlotinib and cetuximab on Calu-3 xenografts

**Figure 7:**
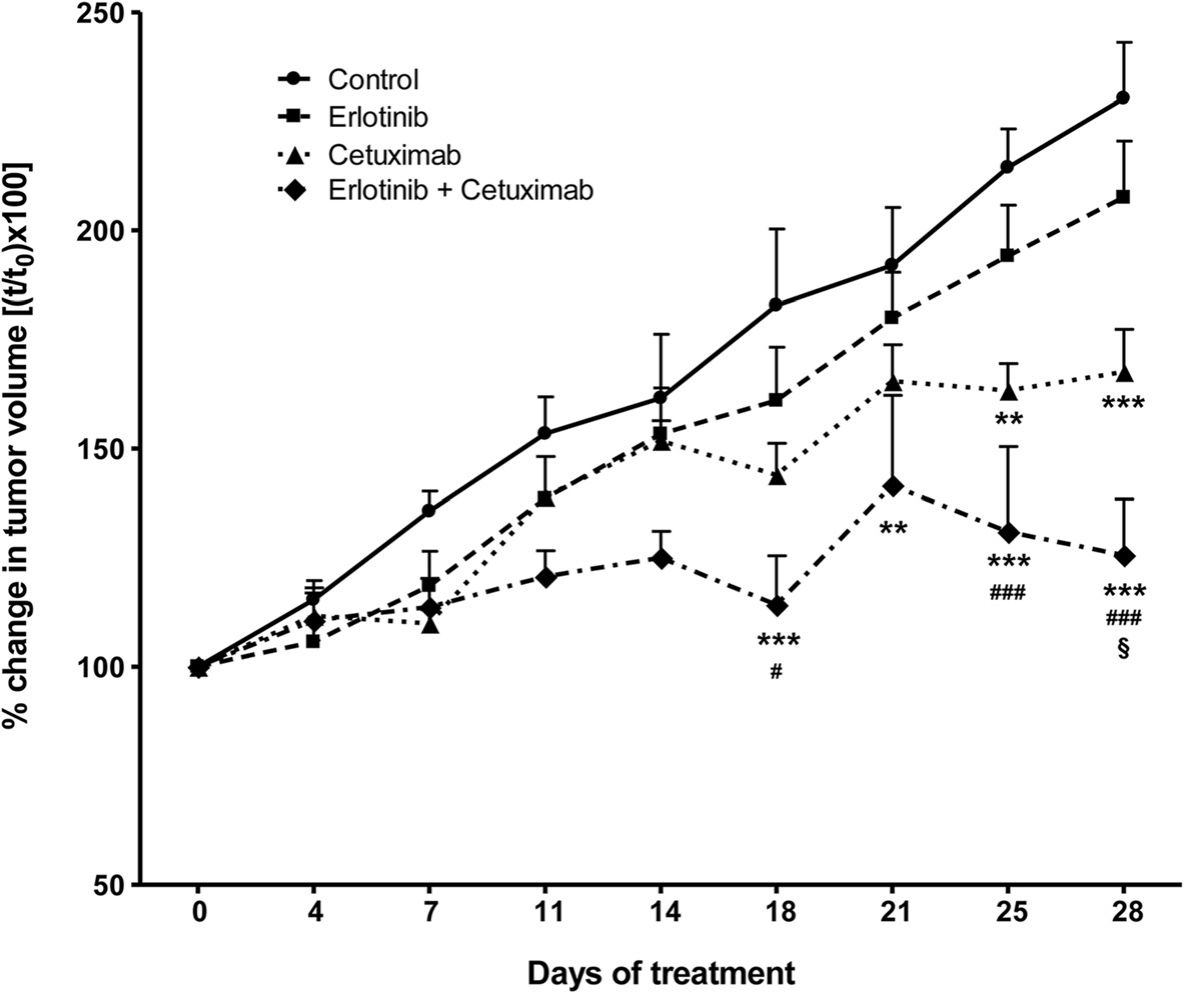
**Antitumour activity of erlotinib and cetuximab on Calu-3 xenografts.** Calu-3 cells were suspended in matrigel and sterile PBS (1:1) and implanted s.c. (right flank) on female BALB/c-Nude mice. Tumours were allowed to establish growth after implantation for 14 days, and the treatments started when tumours reached an average volume of 300 mm^3^. Vehicle, erlotinib (25 mg/Kg, orally 5 days/week), cetuximab (2 mg/Kg i.p. twice weekly), or erlotinib plus cetuximab were administered for the duration of the study. Data are expressed as percent change in tumour volume ± SEM of 6 mice per group. (**p < 0.01, ***p < 0.001 vs control; ^#^p < 0.05, ^###^p < 0.001 vs erlotinib; ^§^p < 0.05 vs cetuximab; two-way ANOVA followed by Bonferroni post-test).

To extend our results *in vivo*, we tested the combination of erlotinib with cetuximab in a Calu-3 xenograft model (Figure 7). When tumours were well established (14 days post-injection, average volume of 300 mm^3^) mice were randomized into four treatment groups receiving erlotinib alone, cetuximab alone, the combination, or vehicles as described in the Methods section. Drug treatments were well tolerated, and no signs of toxicity were detected during the study. The treatment with either erlotinib or cetuximab as single agent delayed tumour growth. However, the significance of the treatment versus the control was observed only with cetuximab as single agent or in combination. Interestingly, the treatment with the combination of erlotinib plus cetuximab significantly inhibited tumour growth when compared to both the single agent treatments.

**Figure 8:**
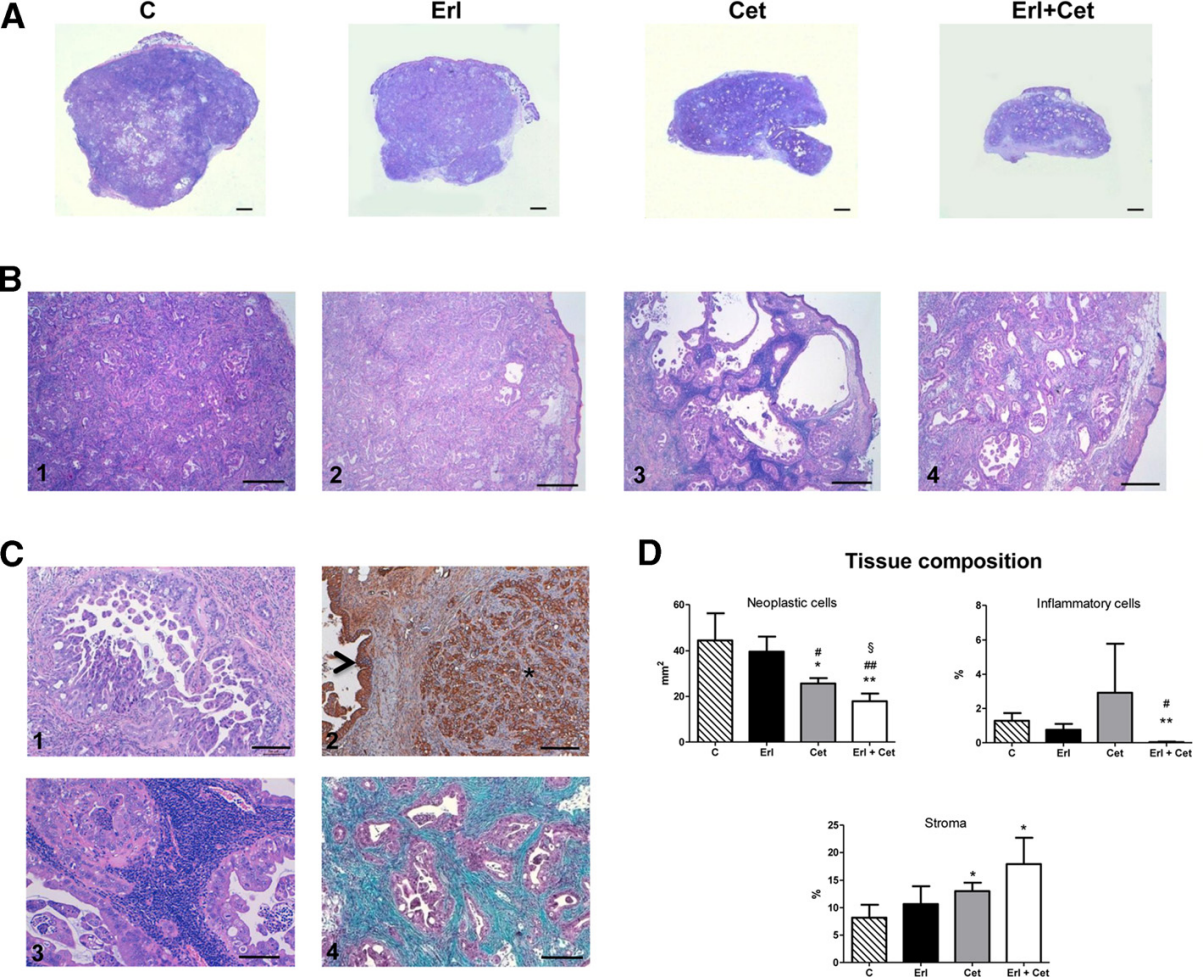
**Hystological analysis of tumours. A**: Selected examples of H&E stained sections of the entire subcutaneous xenografted tumour induced by Calu-3 injection in untreated (C) and erlotinib (Erl), cetuximab (Cet) or erlotinib + cetuximab (Erl + Cet) treated BALB/c nude mice (scale bars: 1 mm). Higher magnification of the same samples are shown on corresponding panels in **B** (scale bars: 500 μm). **C**: representative morphological details of the control neoplastic epithelium (1, H&E) expressing cytokeratin (2,* brown-immunoperoxidase) that also depicts the epidermis (arrowhead). The presence of inflammatory interstitial cells in a cetuximab treated tumour (3, H&E) and the intense collagen deposition (bluish) surrounding neoplastic glands (purple) in a Erl + Cet treated tumour (4, Masson’s trichrome) are shown (scale bars: 100 μm). **D**: Bar graphs illustrating the quantitative measurements of neoplastic, inflammatory cells and stromal compartments composing the tumours. (*p < 0.05, **p < 0.01, vs control; ^#^p < 0.05, ^##^p < 0.01 vs erlotinib; ^§^p < 0.05 vs cetuximab).

The histologic analysis of tumour samples showed that the subcutaneous injection of Calu-3 strikingly reproduced within four weeks the morphological features of human adenocarcinoma (Figures 8A, 8B1-4, 8C-1). Neoplastic epithelial cells clearly expressed cytokeratin (Figure 8C-2) and were organized in secretory glands surrounded by cellularized collagen as evidenced by Masson’s trichrome staining (Figure 8C-4). Regressive phenomena and changes in size of neoplastic glands together with intense stromal reaction were observed in histologic samples of tumours from treated mice. Interestingly, cetuximab clearly resulted in dense inflammatory periglandular infiltrates mostly composed of lymphocytes (Figure 8C-3). Thus, the real impact of treatment on tumour mass within the nodules was assessed by the morphometric analysis of tissue composition. By this quantitative approach, in agreement with gross anatomic measurements, we documented that the combination of erlotinib with cetuximab was the most effective treatment on tumour growth inhibition (Figure 8D).

**Figure 9:**
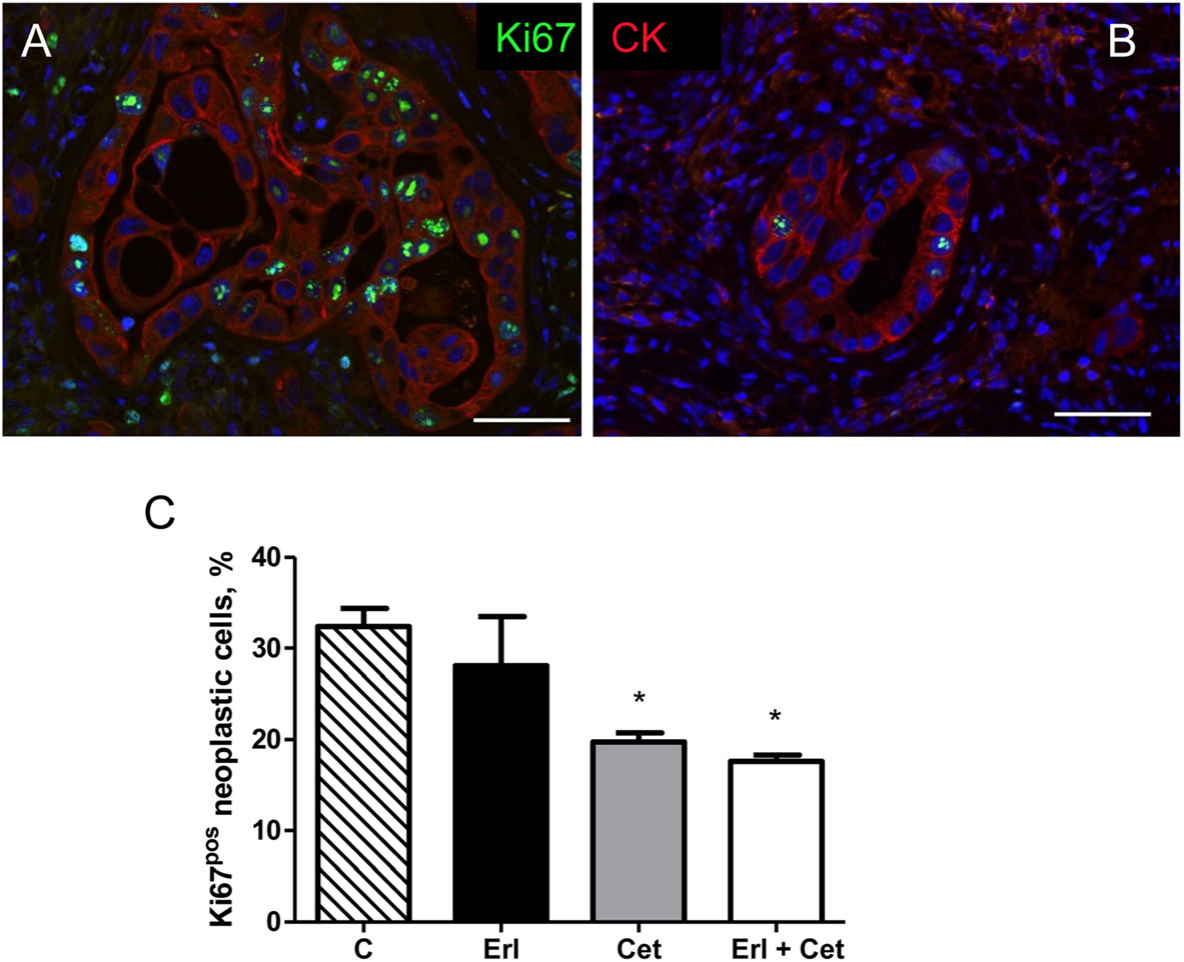
**Immunohistochemical analysis of cellular proliferation.** Immunofluorescence images of Ki67 (green) nuclear labelling of cytokeratin (CK, red) positive neoplastic cells in sections of xenografted tumours from an untreated **(A)** and Erl + Cet treated **(B)** BALB/c nude mouse. **C**: bar graph illustrating the effect of the different treatments on the percentage of cycling (Ki67pos) neoplastic cells within the tumour. CTRL: untreated, ERL: erlotinib, CET: cetuximab, COMB: erlotinib + cetuximab. * p < 0.01 vs CTRL.

This contention was further supported by the immunofluorescence analysis of Ki67 labelling on tumour tissues at the end of the experimental protocol (Figure 9). Erlotinib was able to reduce proliferation of neoplastic cytokeratin^pos^ cells only in association with cetuximab whereas cetuximab had a negative impact on cycling cells also as individual agent. The TUNEL assay indicated that, according with in vitro data, apoptosis was not a significant ongoing cellular event implicated in the effect of different treatments.

We have calculated that 0.026+/−0.016% neoplastic cells were undergoing apoptosis in untreated tumours. Similar low numbers were obtained after Erlotinib or Cetuximab single treatment whereas Erl + Cet increased the amount of TUNEL positive neoplastic cells although reaching a rate of 0.12+/−0.03%. However, we cannot exclude that apoptotic cell death may have contributed to the positive effect of tumor shrinkage at earlier times after drug administration.

Thus, these experimental observations suggest that targeting EGFR by the combination of small molecules and antibodies increases the in vitro and in vivo anti-proliferative activity of both individual agents and seems to be a potent therapeutic strategy against NSCLC.

## Discussion

The potential for dual-agent molecular targeting of the ErbB family, has been clearly demonstrated in pre-clinical models and confirmed on the clinical setting for HER2-targeting agents in breast cancer. However, little is known about this therapeutic strategy for different targets in other tumour types.

In our current study we demonstrated that the combination of erlotinib with cetuximab or trastuzumab may enhance the antitumour activity of EGFR-TKI in NSCLC cell lines harbouring wild-type EGFR and in xenograft models.

The efficacy of the association between an EGFR/HER2 mAbs with TKIs has been documented in preclinical studies in several cell lines originating from different tumour types [15]. In EGFR wild-type H292 and A549 NSCLC cell lines, the combination of either gefitinib or erlotinib with cetuximab was reported to enhance growth inhibition in comparison to single treatment, particularly in the H292 gefitinib sensitive cell line [17]. In the A549 cell line, expressing both EGFR and HER2, the combination of gefitinib with trastuzumab significantly inhibited cell growth and proliferation [18]. In Calu-3 xenograft models, the combined treatment of erlotinib and pertuzumab showed an enhanced antitumour activity [19].

A correlation between cetuximab efficacy and EGFR expression has been reported in preclinical studies [20] and recently confirmed in clinical trials. Thus, the phase III FLEX study involving patients with advanced NSCLC showed a strong correlation between high tumour EGFR overexpression and the efficacy of adding cetuximab to platinum based first-line chemotherapy [12].

The combination of a TKI and a mAb was explored as a potential strategy to overcome acquired resistance to first-generation EGFR-TKIs. Kim and colleagues demonstrated that the combination of lapatinib with cetuximab overcame gefitinib resistance due to the secondary T790M mutation in NSCLC by inducing enhanced cytotoxicity both *in vitro* and *in vivo*[21]. Furthermore, the association of cetuximab with afatinib has been shown to be effective to overcome T790M-mediated drug resistance [22].

However, the combination of erlotinib with cetuximab did not lead to a significant radiological response in NSCLC patients with clinically defined acquired resistance to erlotinib indicating that such strategy is not sufficient to overcome acquired resistance to erlotinib [23].

The mechanisms leading to an enhanced activity of combining a TKI with a monoclonal antibody have been ascribed, in other cancer cell models, either to a more efficient inhibition of TK receptors [17] or to an increased targeted receptors on plasma membrane induced by TKIs [24,25]. Scaltriti et al. showed that lapatinib enhanced the effects of trastuzumab by inducing HER-2 stabilization and accumulation at the cell surface of breast cancer cell lines [24], and Mimura et al. reported that lapatinib induced accumulation of HER-2 and EGFR on esophageal cancer cell lines evoking trastuzumab- and cetuximab- mediated ADCC [25].

ADCC, one of the killing mechanism of the immune system mediated by Natural Killer cells, plays a pivotal role in the anti-cancer effects exerted by mAbs. Therefore, increasing the ADCC activity is an important objective in the development of novel therapeutic approaches.

It has been recently demonstrated that the EGFR inhibitors gefitinib and erlotinib enhance the susceptibility to NK cell mediated lysis of A549, NCI-H23 and SW-900 lung cancer cell lines [26] by the induction of ULBP1 (a ligand of the NK cell activation receptor NKG2D). These data indicate that EGFR blockade could not be the only mechanism of action of EGFR inhibitors in vivo. The efficacy of these inhibitors in lung cancer may be at least in part mediated by increased susceptibility to NK activity. Moreover, cetuximab serves as a potent stimulus for NK functions including INF-gamma production [27] and is also associated with a complement –mediated immune response [28].

We here demonstrated that erlotinib induces an accumulation of EGFR and/or HER2 protein at the plasma membrane level only in TKI sensitive NSCLC cell lines whereas, in resistant cells (both, intrinsic or MET amplification-mediated acquired resistance), this enhancement was not observed. The anti-tumour effect of drug combination was more evident in ADCC experiments compared with cell viability experiments. In the Calu-3 xenograft model, the combined treatment resulted in a lower rate of tumour growth, suggesting the involvement of NK activity as a determinant factor to improve the efficacy of the combined treatment. Moreover, regressive phenomena and changes in size of neoplastic glands together with intense stromal reaction were observed in histologic samples of tumours from mice treated with cetuximab alone or the combination.

The reason why EGFR inhibitors such as gefitinib, erlotinib or lapatinib induce EGFR accumulation only in sensitive cells could be ascribed to their ability to inhibit signal transduction pathways downstream EGFR. The constitutive activation of signaling pathways downstream of EGFR (i.e. presence of RAS mutations) is indeed a recognized mechanism of resistance against reversible EGFR-TKIs [29]. The inhibition of the MAPK pathway might represent a link between EGFR inhibition and EGFR accumulation since U0126, a well known MEK1/2 inhibitor, induced EGFR accumulation in Calu-3 cells, while none of PI3K/AKT/mTOR inhibitors tested was effective. A correlation between MAPK pathway and protein degradation by the ubiquitin system was described for the pro-apoptotic BH3-only protein BIM, indeed in the absence of MAPK activation, BIM protein accumulated in the cell promoting activation of apoptotic cell death [30].

Considering that EGFR TKIs, in particular erlotinib, demonstrated to be effective only in a small percentage of NSCLC patients not harboring *EGFR* mutations, our preclinical results could support clinical trials on the combinations of erlotinib and cetuximab or trastuzumab aiming to improve treatment efficacy. Although the addition of cetuximab to erlotinib is insufficient to overcome erlotinib resistance in *EGFR*-driven lung adenocarcinoma [23], the clinical potential of dual-agent molecular targeting of the EGFR in patients with *EGFR* wild-type tumours remains to be elucidated and may represents an interesting research area to be pursued.

## Conclusions

In this study we explored the potential of combining erlotinib with cetuximab or trastuzumab in improving the efficacy of EGFR targeted therapy in EGFR wild-type erlotinib-sensitive NSCLC cell lines. Our results indicate that erlotinib, through ERK inhibition, increases surface expression of EGFR and/or HER2 only in erlotinib sensitive NSCLC cell lines and in turn leads to increased susceptibility to ADCC both in vitro and in xenografts models.

These data prompt future adequate clinical trials that will give the ultimate proof of the utility of this combined treatment for the care of NSCLC patients carrying EGFR-wild type that are sensitive to TKIs.

## Methods

### Cell culture

The human NSCLC cell lines H322, H292, Calu-3, H1299, A549, H1703 and Calu-1 were obtained from American Type Culture Collection (Manassas, VA, USA) and were cultured as recommended. The PC9, HCC827 and HCC827GR5 cell lines were kindly provided by Dr P. Jänne (Dana-Farber Cancer Institute, Boston MA, USA). All cells were maintained under standard cell culture conditions at 37°C in a water-saturated atmosphere of 5% CO_2_ in air. As previously reported [31] cells showing by proliferation assays IC_50_ for erlotinib < 1 μM were considered sensitive (H322, H292, Calu-3, PC9, HCC827) while cell lines with IC_50_ > 5 μM (H1299, A549, H1703, Calu-1, HCC827GR5) were considered resistant.

### Drug treatment

Erlotinib, gefitinib, cetuximab, trastuzumab and rituximab were from inpatient pharmacy. RAD001, NVP-BKM-120 and NVP-BYL-719 were from Novartis.

Stock solutions of 20 mM drugs were prepared in dimethylsulfoxide (DMSO) (with the exception of mAbs), stored at −20°C and diluted in fresh medium for use. The final concentration of DMSO never exceeded 0.1% v/v.

### Western blot analysis

Procedures for protein extraction, solubilization, and protein analysis by 1-D PAGE are described elsewhere [32,33]. Fifty μg of proteins from lysates were resolved by 7.5% SDS-PAGE and transferred to PVDF membranes. Membranes were incubated with: 1:1000 rabbit polyclonal anti-EGFR; 1:1000 rabbit mAb anti-HER2/ErbB2; 1:1000 rabbit mAb anti-Phospho-p70S6K (Thr421/Ser424); 1:1000 mouse mAb anti-Phospho-p44/42 MAPK (ERK1/2); 1:1000 rabbit mAb anti-p44/42 MAPK (ERK1/2) (Cell Signaling Technology, Beverly, MA, USA); 1:1000 mouse mAb anti- Transferrin Receptor (Invitrogen Corporation, Camarillo, CA, USA); 1:3000 mouse mAb anti-Actin (Sigma–Aldrich, St Louis, MO, USA). Blots were then washed and incubated with HRP-anti-mouse or HRP-anti-rabbit antibodies at 1:20000 dilution (Pierce, Rockford, IL, USA). Immunoreactive bands were visualized using an enhanced chemiluminescence system (Immobilion^TM^ Western Cemiluminescent HRP Substrate, Millipore USA).

### Cell surface protein isolation

Calu-3 cells were grown in T75 flasks and treated with 0.5 μM erlotinib for 24 h. Cells were incubated with EZ-LINK Sulfo-Biotin (Pierce) for 2 h at 4°C with gentle rotation. The reaction was stopped by washing twice with 25 mM Tris–HCl (pH 7.5) in PBS (phosphate-buffered saline) and cells were scraped into ice-cold lysis buffer (50 mmol/l HEPES, pH 7.0, 10% glycerol, 1% TritonX-100, 5 mmol/l EDTA (ethylenediaminetetraacetic acid), 1 mmol/l MgCl_2_, 25 mmol/l NaF, 50 μg/ml leupeptin, 50 μg/ml aprotinin, 0.5 mmol/l orthovanadate, and 1 mmol/l phenylmethylsulfonyl fluoride). Lysates were centrifuged at 15000 g for 20 min at 4°C, and supernatants were removed and assayed for protein concentration using the DC Protein assay (Bio-Rad, CA, USA). A volume of 500 μl of lysis buffer containing equal amount of proteins was incubated with UltraLink Immobilized NeutrAvidin protein (Pierce) for 2 h at 4°C with gentle rotation, washed three times with lysis buffer before suspension in SDS (sodium dodecyl sulfate)-loading buffer and then resolved by SDS-PAGE.

### Flow cytometry

For the determination of EGFR and HER2 protein membrane levels, NSCLC cell lines H322, Calu-3 and H292 were treated with 1 μM erlotinib for 24 h. One million cells per condition were then incubated with Isotype control Monoclonal Mouse IgG1/R-PE (Ancell IRP, Bayport, MN, USA), PE mouse anti-Human EGFR (Calu-3 and H322 cells) (BD Biosciences, San Josè, CA, USA) or PE mouse anti-Human HER2 (H322 and H292) (BD Biosciences). After the incubation the analysis was performed with an EPICS-XL flow cytometer.

For the relative quantization of EGFR or HER2 binding sites, NSCLC cell lines H322, Calu-3, H292 were treated with 1 μM erlotinib for 24 h. One million cells were then dispensed for each condition and treated with either 20 μg/ml rituximab (Isotype control), cetuximab (Calu-3 and H322) or trastuzumab (H292) for 1 h. After the incubation with PE-anti-human-IgG (BD Biosciences), the analysis was performed with an EPICS-XL flow cytometer.

The values of mean fluorescence intensity (MFI) were converted in units of equivalent fluorochrome (MEF) using the FluoroSpheres 6-Peak Kit (Dako, CA, USA).

### Quantitative real-time PCR

Total RNA was isolated by the TRIzol® reagent (Invitrogen, Carlsbad, CA, USA) and reverse transcribed as previously described [33].

The transcript levels of *EGFR* gene were assessed by Real-Time qRT-PCR on an iCycler iQ Multicolor RealTime PCR Detection System (Bio-Rad, Hercules, CA, USA).

Primers and probes included: EGFR-F (5′-GCCTTGACTGAGGACAGCA-3′), EGFR-R (5-TTTGGGAACGGACTGGTTTA-3), EGFR-probe (5′-FAM CTTCCTCC3′DQ); PGK1-F (5′-GGAGAACCTCCGCTTTCAT-3′), PGK1-R (5′-CTGGCTCGGCTTTAACCTT-3′), PGK1-probe (5′-FAM GGAGGAAG 3′DQ); RPL13-F (5′-ACAGCTGCTCAGCTTCACCT-3′), RPL13-R (5′-TGGCAGCATGCCATAAATAG-3′), RPL13-probe (5′-FAMCAGTGGCA3′DQ); HPRT-F (5′-TGACCTTGATTTATTTTGCATACC-3′), HPRT-R (5′CGAGCAAGACGTTCAGTCCT-3′), HPRT-probe (5′-FAM GCTGAGGA 3′DQ).

The amplification protocol consisted of 15 min at 95°C followed by 40 cycles at 94°C for 20s and at 60°C for 1 min.

The relative transcript quantification was calculated using the *geNorm* algorithm for Microsoft Excel^TM^ after normalization by expression of the control genes [*phosphoglycerate kinase1* (*PGK1*), *ribosomal protein L13* (*RPL13*) and *hypoxanthine*-*guanine*-*phosphoribosyltransferase* (*HPRT*)] and expressed in arbitrary units (a.u.).

### MTT assay

The cells were seeded into 96-well plate in quadruplicate and were exposed to various treatments. After 96 h, 100 μl of 3-(4,5-dimethylthiazol-2-yl)-2,5 diphenyltetrazolium bromide (MTT) solution (1 mg/ml, Sigma-Aldrich) was added to each well and incubated. After 4 h, crystalline formation was dissolved with DMSO and the absorbance at 570 nm was measured using the microplate-reader 550 (BioRad).

### Isolation and culture of NK cells

Human PBMC were isolated from buffy coat of healthy donors by using a Lympholyte-H density gradient centrifugation (Cederlane Burlington, Ontario, Canada). Highly purified CD56^+^ natural killer (NK) cells were obtained by magnetic separation using the NK Cell Isolation Kit and the autoMACS Separator (Miltenyi Biotec, Cologne, Germany) according to the user manual.

Purified NK cells were resuspended in culture medium (RPMI 1640 without phenol red and supplemented with heat inactivated 10% FCS, 50 U/ml penicillin, 50 U/ml streptomycin, 2 mmol/l glutamine) plated and preincubated at 37°C for up to 18 h in the presence of human Interleukin-2 (IL-2, 100 U/ml, Miltenyi Biotec).

### ADCC assay

Antibody-dependent cell-mediated cytotoxicity (ADCC) was measured with the CytoTox 96 non-radioactive cytotoxicity assay (Promega, Madison, WI, USA) according to manufacturer’s instructions. 2x10^3^ Calu-3, H322, H292 or H1299 cells were treated for 24 h with 1 μM erlotinib, and then seeded with purified NK cells (ratio of 1:25 and 1:50) in a 96-well plate and incubated with 10 μg/ml cetuximab or trastuzumab. After 4 hours the lactate dehydrogenase (LDH) release was determined and the percentage of cytotoxicity was calculated after correcting for background absorbance values according to the following formula:

(1) $$\mathrm{\%Cytotoxicity}=\frac{\mathrm{Experimental}-\mathrm{Effector}\;\mathrm{spontaneous}-\mathrm{Target}\;\mathrm{spontaneous}}{\mathrm{Target}\;\mathrm{maximum}-\mathrm{Target}\;\mathrm{spontaneous}}\times 100$$

### Tumour xenografts

All experiments involving animals and their care were performed with the approval of the Local Ethical Committee of University of Parma, in accordance with the institutional guidelines that are in compliance with national (DL116/92) and international (86/609/CEE) laws and policies. Twenty-four Balb/c-Nude female mice (Charles River Laboratories, Calco, Italy) were housed in a protected unit for immunodeficient animals with 12-hour light/dark cycles and provided with sterilized food and water *ad libitum*. At the time of xenograft establishment, mice were 8 weeks old and weighted ~20g. 200 μl of matrigel (BD Biosciences) and sterile PBS (1:1) containing 1x10^7^ Calu-3 cells, were subcutaneously injected on the right flank of each mouse (using a 1 ml syringe, needle G25). When tumour volume reached an average size of 300 mm^3^, 14 days after injection, animals were randomized into four groups and the treatment started. After 4 weeks, mice were euthanized by cervical dislocation and tumours collected for immunohistochemistry and histological analysis.

Erlotinib (25 mg/Kg) was administered orally in 1% methylcellulose, 0.2% Tween 80 in sterilized water 5 days/week. Cetuximab (2 mg/Kg) was intraperitoneally injected in sterile saline solution 2 days/week. Control group received both oral gavage of 1% methylcellulose, 0.2% Tween 80 in sterilized water 5 days/week and i.p. injection of sterile saline solution (0.9%) 2 days/week.

Dosages of drugs were chosen halving the one used in a previous study in NSCLC-xenograft models, in order to avoid the complete inhibition of tumour growth by the single agent treatment and to better highlight the effect of erlotinib-cetuximab combination [19,34].

Tumour xenografts were measured twice a week, tumour volume was determined using the formula: (length x width^2^)/2. Final data are expressed as percent of volume increase: (tumour volume/pre-treatment tumour volume) x 100.

### Morphometric and immunohistochemical analysis of tumour xenografts

Formalin fixed samples were embedded in paraffin. From each tumour serial sections of 5 μm thickness were obtained and stained with Haematoxylin and Eosin (H&E), Masson’s Trichrome and for immunohistochemistry. Morphometric analysis was performed in order to evaluate: (a) the numerical density of neoplastic cells, (b) the volume fraction of interstitial inflammatory cells, (c) the volume fraction of fibrosis and (d) the fraction of proliferating and apoptotic cells.

In particular, for each section stained with H&E, a quantitative evaluation of tissue composition was performed. To better define the fraction occupied by neoplastic and non neoplastic cells, sections were stained with pancytokeratin antibodies (monoclonal mouse, 1:500, o.n. 4°C, Dako) revealed through biotin-streptavidin-DAB system, as repeatedly described. The numerical density (n/mm^2^) of pancytokeratin positive neoplastic cells was computed.

In addition, cell proliferation and apoptotic death were investigated by fluorescence microscopy. Thus, Ki67 labeling (rabbit polyclonal antibody, Vector) and the Terminal deoxynucleotidyltransferase (TdT)–mediated dUTP nick end labeling (TUNEL) assay (Roche Diagnostics, Italy) on cytokeratin^pos^ neoplastic cells were revealed by specific fuorescent probes.

The area occupied by interstitial cells was expressed as percentage of the total area explored. By the same approach, the volume fraction of fibrosis was calculated on Masson’s Trichrome stained sections. To define the volume fractions, the number of points overlying each tissue components was counted and expressed as percentage of the total number of points explored.

All these morphometric measurements were obtained with the aid of a grid defining a tissue area of 0.23 mm^2^ and containing 42 sampling points each covering an area of 0.0052 mm^2^.

All these evaluations were performed on the entire section of each tumour sample of each experimental group of animals using an optical microscope (250X final magnification).

### Statistical analysis

Statistical analyses were carried out using GraphPad Prism version 5.0 software (GraphPad Software Inc., San Diego, CA, USA). Results are expressed as mean values ± standard deviations (SD) for the indicated number of independent measurements. Differences between the mean values recorded for different experimental conditions were evaluated by Student’s t-test, and P values are indicated where appropriate in the figures and in their legends. A P value <0.05 was considered as significant.

For in vivo studies comparison among groups was made using analysis of variance (two-way ANOVA repeated measures) followed by Bonferroni’s post-test. Analysis was performed using Prism 5.0 (GraphPad Software) and differences were considered significant when P value was below 0.05. The nature of the interaction between erlotinib and cetuximab was calculated using the Bliss interaction model [35].

## Abbreviations

ADCC: Antibody-dependent cellular-cytotoxicity; EGFR: Epidermal growth factor receptor; MAPK: Mitogen-activated protein kinase; NSCLC: Non small cell lung cancer; TKI: Tyrosine kinase inhibitor.

## Misc

Andrea Cavazzoni and Roberta R Alfieri contributed equally to this work.

## Competing interest

All authors declare that they have no competing interests.

## Authors’ contribution

AC carried out ADCC experiments, interpreted the results and assisted with the draft of the manuscript; DC isolated and cultured NK cells and carried out flow cytometry analysis; FS, LA and PC performed the *in vivo* studies; PM carried out RT-PCR experiments; MG and MB carried out Western blot analysis; EG performed the statistical analysis, SLM and CF carried out cell growth experiments; FQ, GG and DM carried out immunohistochemical analysis; AM, MT, EB and PGP critically revised the manuscript; AA designed the project and assisted with the draft of the manuscript; RRA, analyzed the results and wrote the manuscript. All authors read and approved the final manuscript.
